# Detection of Volatile Sulfur Compounds in Mangoes Using Sorptive Extraction Methods

**DOI:** 10.3390/molecules31132276

**Published:** 2026-06-29

**Authors:** Zhibo Li, Yantong Zheng, Yunle Huang, Christina Shu Min Liew, Lingyi Li, Kim Huey Ee, Rui Min Vivian Goh, Shanbo Zhang, Lionel Jublot, Shao Quan Liu, Bin Yu

**Affiliations:** 1 Department of Food Science and Technology, National University of Singapore, S14 Level 5, Science Drive 2, Singapore 117542, Singapore; zhibo@u.nus.edu (Z.L.); e1350676@u.nus.edu (Y.Z.); e0729493@u.nus.edu (L.L.); e0729795@u.nus.edu (S.Z.); 2 Mane SEA Pte Ltd., 3 Biopolis Drive, #07-17/18/19 Synapse, Singapore 138623, Singapore; yunle.huang@mane.com (Y.H.); ee.kim-huey@mane.com (K.H.E.); vivian.goh@mane.com (R.M.V.G.); lionel.jublot@mane.com (L.J.); 3 Gerstel LLP, 10 Science Park Road, 02-18 The Alpha, Singapore 117684, Singapore; christina_liew@gerstel.com

**Keywords:** mango, volatile sulfur compounds, sorptive extraction, HS-TFSPME-SBSE, HiSorb

## Abstract

Volatile sulfur compounds (VSCs) are important contributors to mango volatile profiles but are challenging to analyze due to their trace concentrations, susceptibility to transformation, and interference from the complex fruit matrix. This study investigated how key extraction parameters—material chemistry, surface area-to-volume ratio, sorbent volume, temperature, and time—affect VSC extraction using mango as a complex botanical model. Three sorptive extraction configurations were evaluated: HiSorb (PDMS and DVB/CWR/PDMS) and a high-capacity combined headspace thin-film solid-phase microextraction and stir bar sorptive extraction (HS-TFSPME-SBSE) system. The optimized HS-TFSPME-SBSE configuration (40 °C, 150 min) provided the broadest VSC coverage, achieving limits of detection of 0.1–0.6 μg/kg and good linearity (R^2^ > 0.9927). In spiked mango puree (10 μg/kg), HS-TFSPME-SBSE detected methyl mercaptan and diallyl trisulfide, which were not recovered using HiSorb (PDMS). Application to three mango cultivars (Golden Honey, Sindhura, and Palmer) revealed broader VSC profiles and enabled differentiation of cultivars and tissues (flesh and peel) through principal component analysis (PCA). Distinct cultivar-associated VSC patterns were observed, including elevated dimethyl disulfide in Golden Honey and ethyl 3-(methylthio)-*cis*-2-propenoate in Sindhura. These findings demonstrate the suitability of HS-TFSPME-SBSE for sensitive profiling of trace VSCs in complex fruit matrices.

## 1. Introduction

Mango (*Mangifera indica* L.) is one of the most widely cultivated and commercially important tropical fruits worldwide [1,2]. Mangoes are consumed both fresh and in various processed forms such as juices, purees, jams, and dried fruits. Additionally, mango byproducts, such as peel and kernel, have found increasing use in nutraceutical products [3,4]. Its popularity is largely attributed to its characteristic flavor, which arises from a complex mixture of volatile and non-volatile compounds. Major volatile classes such as terpenes, esters, aldehydes, and ketones have been extensively studied and are known to contribute to the distinctive aroma profiles of different mango cultivars [5,6,7,8].

While extensive studies have elucidated these major volatile constituents, less under-stood are the trace molecules, many of which are equally important to the odor profile of mango [9]. One chemical class of compounds that falls into this category includes volatile sulfur compounds (VSCs) [10]. VSCs contributing to mango flavor have been relatively less explored, despite emerging evidence indicating their crucial role in tropical fruit aroma. These VSCs, albeit their low concentrations, can exert a strong odor impact due to their low sensory detection thresholds, contributing to the characteristic juicy, fresh, and authentic aroma of mangoes [11].

Given their sensory importance, accurately identifying and quantifying VSCs is essential for characterizing mango aroma and assessing fruit quality. However, the analysis of these compounds in mango presents formidable analytical challenges. One main challenge faced is the presence of VSCs at trace level, requiring extraction and detection techniques with exceptional sensitivity [10]. In addition, the mango matrix is highly complex, containing high concentrations of sugars, organic acids, and a wide range of non-sulfur volatile organic compounds (VOCs), which can lead to matrix effects such as signal sup-pression and co-elution during chromatographic separation, thereby necessitating high method selectivity [12]. Furthermore, many VSCs exhibit diverse physicochemical proper-ties and may be prone to losses during sample handling due to their volatility [10]. While additional challenges such as the presence of glycosidically bound precursors, compound instability (e.g., oxidation of thiols), and potential artifact formation during analysis have been reported in the literature [13,14,15,16], these aspects were not specifically investigated in the present study. Instead, this work examines the application of sorptive extraction methods for the detection of trace flavour-impactful VSCs in mango, with attention to challenges arising from complex matrices, ultra-trace concentrations, and general analyte instability.

Conventional techniques of volatile analysis often struggle to meet these demanding requirements. Traditional solvent extraction methods lack sufficient enrichment of trace compounds, while basic solid-phase microextraction (SPME) can be limited by its sorbent volume and sensitivity for trace analytes [17,18]. Moreover, SPME fibers are prone to competitive displacement among VSCs, where heavier analogs displace low-molecular-weight thiols from limited active sites, causing erratic calibration [19]. As an advancement, thin-film solid-phase microextraction (TFSPME) employs coatings with greater surface areas compared to standard fibers, which can improve extraction kinetics and potentially mitigate competitive adsorption effects in complex matrices [20,21,22]. Another widely utilized approach, stir-bar sorptive extraction (SBSE), can significantly enhance analyte enrichment through 50–250 times larger extraction phase and is more robust than SPME [23,24]. However, SBSE relies solely on polydimethylsiloxane (PDMS) coating, which are non-polar and give poor recovery for polar VSCs, while alternative polar coatings are in-compatible with thermal desorption and suffer from poor stability [25,26].

In recent years, high-capacity sorptive extraction probes like HiSorb have found ap-plications in flavor analysis, offering considerably larger sorbent volumes and surface areas than SPME and SBSE, alongside a wider selection of sorbent phase chemistries which enhance the range of analytes detected [27,28]. This increased phase volume allows for greater analyte enrichment and can reduce competitive adsorption effects, thereby enabling more efficient recovery of low-abundance compounds. Combining multiple extraction devices such as TFSPME and SBSE allows for the integration of the large sorptive capacity of SBSE with the high surface-area-to-volume ratio of TFSPME to enhance both ex-traction efficiency and sensitivity for a wide range of volatiles, achieving a broader linearity range, improved sensitivity, and higher recoveries [22,29,30]. Specifically, the combined headspace thin-film solid-phase microextraction- stir bar sorptive extraction (HS-TFSPME-SBSE) method developed by Huang et al. (2020) [29] achieved up to 20% higher recoveries and extended linearity ranges by 2–3 orders of magnitude compared to single techniques, with good repeatability (relative standard deviation, RSD < 13.1%). These latest advancements indicate significant potential for tackling the intricate challenges of trace VSC analysis. Although high-capacity sorptive extraction approaches such as HiSorb and HS-TFSPME-SBSE have demonstrated advantages for volatile analysis, their suitability for the extraction of trace VSCs in complex fruit matrices has not been systematically evaluated. This knowledge gap is particularly relevant given the analytical challenges associated with sulfur-containing volatiles and their importance to fruit aroma.

Therefore, the objective of this study was to investigate the performance of different sorptive extraction techniques for the detection of VSCs in the complex mango matrix and to provide analytical insights into factors influencing trace-level flavour compound analysis in complex food systems. The study focused on evaluating the capability of these methodologies to enrich and detect trace-level VSCs in a complex mango matrix and to compare their analytical performance in terms of extraction efficiency, sensitivity, linearity, and reproducibility. Specifically, the study aimed to (1) investigate the influence of key extraction parameters using VSC-spiked mango puree as a model matrix, (2) compare extraction methods with respect to sensitivity, extraction efficiency, linearity, and reproducibility, and (3) demonstrate the applicability of the optimized method through profiling of VSCs in different mango cultivars and tissues. By focusing on trace sulfur-containing volatiles, this work provides methodological insights for the analysis of challenging aroma compounds in complex food matrices.

## 2. Results and Discussion

### 2.1. Comparison of Extraction Efficiencies Among Different Sorptive Techniques

To evaluate the extraction performance of different sorptive techniques for VSCs, six headspace sampling methods were tested using mango puree spiked with 5 mg/kg of each VSC: HS-stir bar sorptive extraction (HS-SBSE), HiSorb (PDMS and divinylbenzene/carbon wide range/PDMS (DVB/CWR/PDMS)), HS-thin-film solid-phase microextraction (HS-TFSPME), HS-TFSPME combined with one twister bar (HS-TFSPME-SBSE), and HS-TFSPME combined with two twister bars (HS-TFSPME-SBSE × 2). All extractions were performed at 40 °C for 150 min, a temperature selected based on previous studies demonstrating its effectiveness for VSCs extraction in complex food matrices [25,31,32]. Peak areas were used to assess extraction efficiency and relative standard deviation (RSD) for evaluation of method repeatability.

This relatively high spiking concentration (5 mg/kg) was intentionally employed during this initial comparative screening phase to ensure that all target VSCs, across their varying volatilities and affinities for the sorbents, would yield robust and clearly distinguishable analytical signals, well above background noise and potential matrix interferences. This approach would enable a more reliable assessment of the relative extraction efficiencies of the different techniques (based on peak area comparison).

**Figure 1:**
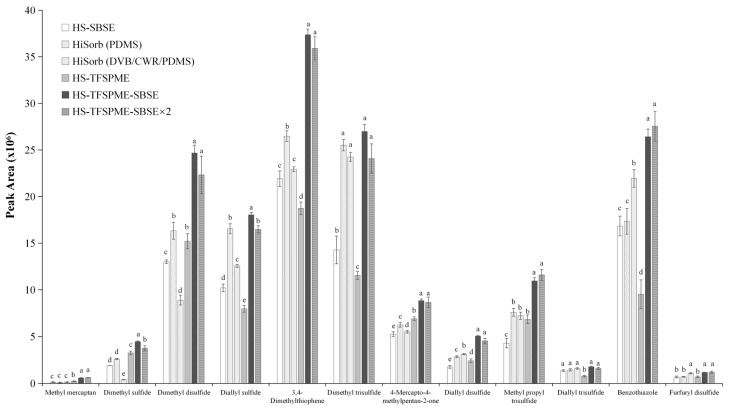
Extraction profiles of 12 volatile sulfur compounds (VSCs) spiked in mango puree using HS-SBSE, HiSorb (PDMS), HiSorb (DVB/CWR/PDMS), HS-TFSPME, HS-TFSPME-SBSE and HS-TFSPME-SBSE × 2 (40 °C, 150 min). Different lower-case letters above the bars denote that values are significantly different (*p* < 0.05) within the compound group.

As shown in Figure 1, HS-TFSPME-SBSE exhibited the highest overall extraction efficiency. For most of the 12 VSCs, it achieved the largest peak areas, including dimethyl disulfide, 3,4-dimethylthiophene, dimethyl trisulfide, benzothiazole, and furfuryl disulfide, spanning a wide range of molecular weights and polarities. Combining the TFSPME device and twister bar sorption phases enabled dual pathways for analyte recovery across polar and non-polar domains. This synergy enhanced sensitivity and dynamic range, as previously reported for volatile compounds [29].

In comparison, when only single device was used in the case of HS-SBSE and HS-TFSPME, limited performance, yielding lower peak areas and narrower analyte coverage was observed. While HS-TFSPME demonstrated moderate efficiency for low molecular weight analytes such as dimethyl sulfide, it was clearly outperformed by the combination technique in terms of both intensity and compound range. Therefore, HS-SBSE and HS-TFSPME were excluded from further optimization.

Contrary to expectations, HS-TFSPME-SBSE × 2 (two twister bars) did not significantly improve extraction efficiency over HS-TFSPME-SBSE (single twister bar). For dimethyl sulfide and 3,4-dimethylthiophene, peak areas were significantly reduced. The finding aligns with a previous study by Marín-San Román et al. (2022) [33]. This was possibly due to competitive sorbent dynamics or excessive phase loading, which could hinder analyte transfer [25]. Given the increased material involved and added handling complexity, HS-TFSPME-SBSE × 2 was not considered further.

HiSorb (PDMS) and HiSorb (DVB/CWR/PDMS) showed moderate to strong performance for some analytes but generally failed to reach the same peak areas as HS-TFSPME-SBSE. While HiSorb (PDMS) performed reasonably well for diallyl trisulfide and dimethyl trisulfide, its overall extraction capacity was clearly inferior to HS-TFSPME-SBSE. Similarly, the DVB/CWR/PDMS composite coating, though designed to provide broader polarity coverage, did not outperform PDMS or the HS-TFSPME-SBSE under the current extraction conditions. When the triple phases are blended at a fixed total coating volume, the effective PDMS phase volume is expected to decrease, directly reducing its absolute adsorption capacity for PDMS-affinitive analytes. In contrast, when the different devices were combined in HS-TFSPME-SBSE, there was no compromise on the amount of sorbent materials, thereby maintaining full sorbent availability per unit volume.

Based on this preliminary screening, HiSorb (PDME), HiSorb (DVB/CWR/PDMS) and HS-TFSPME-SBSE were selected for further investigation. Across all retained methods, RSD values were within acceptable ranges (<15%) for most analytes. The extraction temperature and extraction time of these three methods were then further optimized.

### 2.2. Optimization of HiSorb and HS-TFSPME-SBSE

#### 2.2.1. Extraction Temperature

Optimization of extraction temperature is crucial in sorptive extraction methods when targeting trace-level VSCs in complex fruit matrices like mango. VSCs are thermally labile and susceptible to oxidative degradation or artifact formation, necessitating careful temperature control to ensure analyte stability and accurate profiling. Previous studies on sorptive extraction methods of volatiles in juices have highlighted the importance of optimizing temperature. For instance, study in fruit juice demonstrated that extraction at 40 °C had higher enrichment efficiencies for volatiles compared to room temperature [34]. Similarly, research on VSCs in spirits identified 35 °C as an optimal temperature to balance recovery with minimal thermal degradation [35]. These findings underscore the need for moderate temperature use during extraction.

The effect of extraction temperature on VSC recovery was investigated by comparing extraction performance at 30 °C and 40 °C using mango puree spiked with 5 mg/kg of VSCs. The results indicate that extraction temperature do influenced VSC recovery.

**Figure 2:**
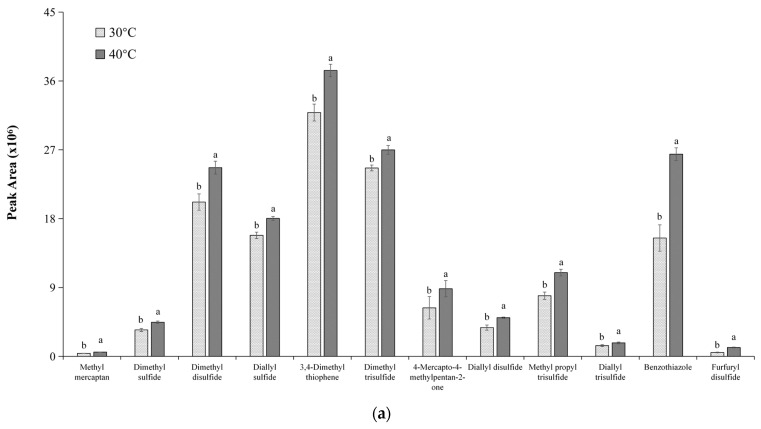

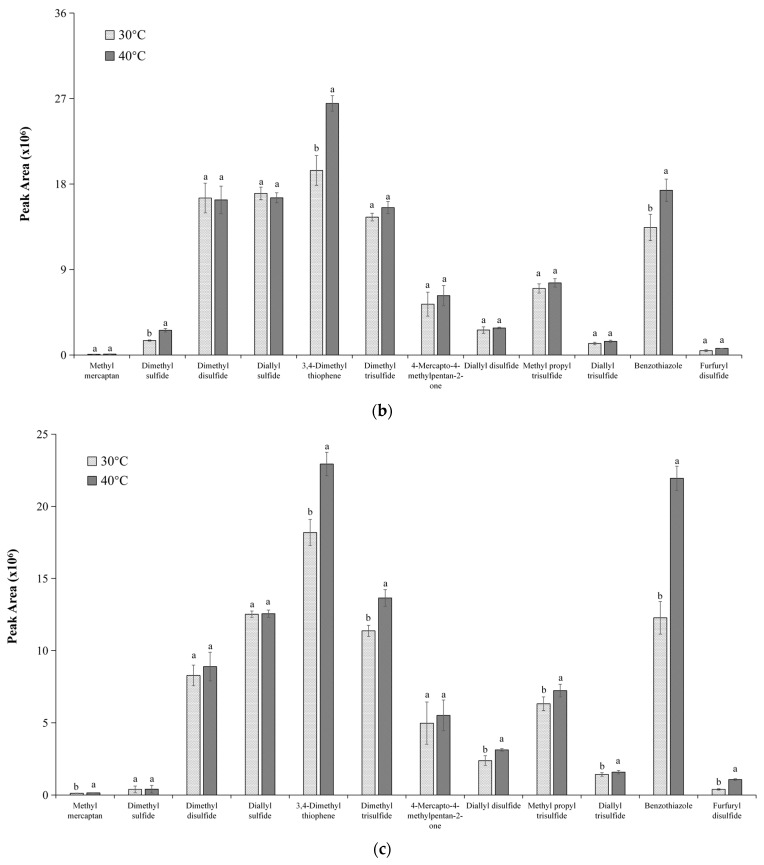
(**a**). Extraction profiles of selected VSCs spiked in mango puree using HS-TFSPME-SBSE (150 min) under different extraction temperatures. Different lower-case letters above the bars denote that values are significantly different (*p* < 0.05) within the compound group. (**b**). Extraction profiles of selected VSCs spiked in mango puree using HiSorb (PDMS) (150 min) under different extraction temperatures. Different lower-case letters above the bars denote that values are significantly different (*p* < 0.05) within the compound group. (**c**). Extraction profiles of selected VSCs spiked in mango puree using HiSorb (DVB/CWR/PDMS) (150 min) under different extraction temperatures. Different lower-case letters above the bars denote that values are significantly different (*p* < 0.05) within the compound group.

A general trend of increased VSC extraction efficiency with increased temperature was observed across all three sorptive extraction methods (Figure 2a–c) when the temperature was increased from 30 °C to 40 °C. This enhancement is consistent with the principle that higher temperatures promote VSC partitioning from the sample matrix to the extraction phase [29,31]. Furthermore, the RSDs of VSC measurements were significantly lower at 40 °C than at 30 °C, demonstrating that extraction at the higher temperature led to more consistent and reliable results. The improved precision at 40 °C suggests that the higher temperature may facilitate more efficient and reproducible mass transfer of VSCs to the sorbent phase.

However, VSCs are known to be susceptible to thermal oxidation and volatilization [16]. Therefore, extraction temperatures above 40 °C were not investigated in this study, as the objective was not to maximize volatilization through elevated thermal conditions, but rather to obtain a VSC profile that more closely reflects the aroma composition perceived during normal mango consumption. Since mangoes are typically consumed at ambient or chilled conditions rather than at elevated temperatures, the selected extraction range was considered more representative of the fruit’s sensory profile. This approach aimed to balance efficient VSC extraction with the need to minimize thermally induced analyte degradation, volatilization losses, and the generation of potential extraction artifacts, thereby providing a flavour profile more relevant to realistic sensory perception.

In summary, 40 °C was selected as the optimal extraction temperature for subsequent experiments, based on its superior extraction efficiency, improved precision, and minimal risk of VSC degradation.

#### 2.2.2. Extraction Time

Apart from extraction temperature, optimization of extraction time is equally critical. Excessive extraction time can increase the risk of analyte losses. Previous studies emphasized the need for time optimization. Studies on fruit juice analysis using sorptive extraction methods have shown that an extraction time of 120 min is typically required to reach equilibrium conditions and obtain higher recovery of volatile analytes [34]. These results highlight the importance of selecting the suitable extraction time for detecting trace VSCs.

**Figure 3:**
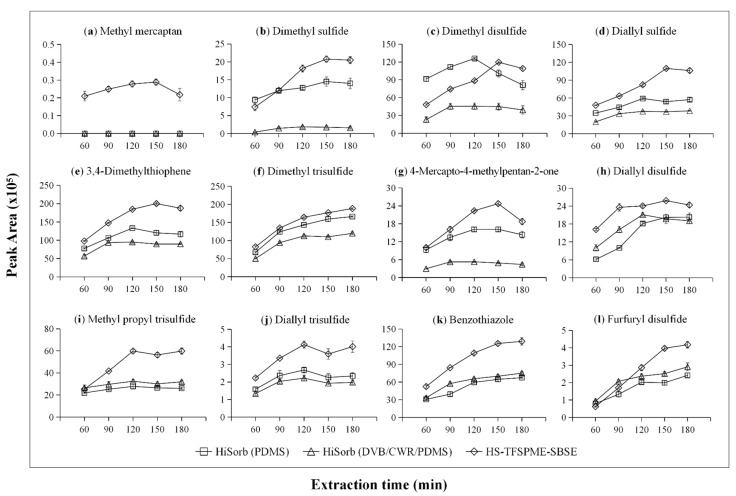
Extraction profiles of selected VSCs spiked in mango puree using HiSorb (PDMS), HiSorb (DVB/CWR/PDMS) and HS-TFSPME-SBSE (40 °C) under different extraction time: (**a**) Methyl mercaptan; (**b**) Dimethyl sulfide; (**c**) Dimethyl disulfide; (**d**) Diallyl sulfide; (**e**) 3,4-Dimethylthiophene; (**f**) Dimethyl trisulfide; (**g**) 4-Mercapto-4-methylpentan-2-one; (**h**) Diallyl disulfide; (**i**) Methyl propyl trisulfide; (**j**) Diallyl trisulfide; (**k**) Benzothiazole and (**l**) Furfuryl disulfide.

In this study, extraction time was optimized for three selected techniques: HiSorb (PDMS), HiSorb (DVB/CWR/PDMS), and HS-TFSPME-SBSE, using 12 VSCs spiked into mango puree at 500 μg/kg. The extraction temperature was maintained at 40 °C. Agitation was intentionally omitted to prevent the oxidation of specific VSCs and avoid artifacts formation. This is so as the stirring process can result in incorporation of oxygen into the sample and disruption of cellular compartments (in mango fruit sample). This can enhance the exposure of thiols in the samples to oxidative enzymes and reactive oxygen species. Hence, resulting in potential catalytic oxidation of these thiols. The effect of extraction time (60, 90, 120, 150, and 180 min) on the extraction profiles of 12 VSCs is illustrated in Figure 3.

For the HiSorb method, both probes demonstrated faster extraction kinetics than HS-TFSPME-SBSE, typically reaching peak area maxima or plateaus at around 120 min. For example, using HiSorb (PDMS), key analytes like dimethyl sulfide (b), dimethyl disulfide (c), and diallyl disulfide (h) showed optimal responses at 120 min, with negligible increases or even slight decreases at 150 or 180 min. Similar trends, albeit with lower overall signals, were observed for HiSorb (DVB/CWR/PDMS). Extending the time beyond 120 min offered little advantage in terms of sensitivity and potentially increased variability for certain analytes. Thus, 120 min was established as the optimal extraction time.

For the HS-TFSPME-SBSE method, the peak areas for the most VSCs exhibited a significant increase, reaching a maximum or near-maximum at around 150 min. While extending the extraction time to 180 min yielded minor increases for less volatile compounds, such as benzothiazole (k) and furfuryl disulfide (l), the overall improvement was negligible. Furthermore, extending the extraction beyond 150 min resulted in an observable increase in RSD for several analytes, potentially indicative of enhanced competition, matrix interference, or desorption/re-equilibration. Therefore, 150 min was selected as the operating condition for subsequent experiments as a practical compromise between extraction efficiency and analysis throughput, rather than as confirmation of equilibrium extraction for all analytes.

Beyond optimization of extraction time, the results also provided insights into the comparative extraction efficiency and characteristics of the methods. As clearly visible in Figure 3, HS-TFSPME-SBSE consistently delivered substantially higher peak areas for nearly all target VSCs compared to both HiSorb probes. This superior extraction capacity may be attributed to the synergistic effect of combining the high surface area and potentially mixed-mode sorption of TFSPME with the large sorbent volume of SBSE, facilitating more comprehensive enrichment across the diverse chemical properties of the VSCs. Comparing the HiSorb probes, HiSorb (PDMS) outperformed HiSorb (DVB/CWR/PDMS) significantly for most VSCs studied here. The lower efficiency of the DVB/CWR/PDMS phase for these analytes suggests that its potential advantage for polar compounds did not translate to better overall performance for this specific group of VSCs, possibly due to unfavorable partitioning or competitive adsorption from the matrix [36,37].

Furthermore, analyte-specific interactions revealed notable differences between the methods. Notably, HS-TFSPME-SBSE reliably detected methyl mercaptan (a) at an initial concentration of 500 µg/kg, whereas HiSorb failed to detect this compound. Simultaneously, dimethyl disulfide (c), a known oxidation product of methyl mercaptan (a) [38,39], exhibited a comparatively high initial signal with HiSorb (PDMS). This observation may indicate transformation of methyl mercaptan (a) during extraction or thermal desorption. Similar sulfur compound interconversions, including oxidation of thiols to disulfides and subsequent sulfur rearrangements, have previously been reported during thermal desorption and hot GC injection conditions, particularly for reactive sulfur-containing analytes [16,38,39]. However, such transformations were not directly investigated in the present study, and alternative explanations, including differences in extraction efficiency, partitioning behavior, analyte stability, or desorption characteristics between the two techniques, cannot be excluded. These observed differences may also be influenced by the distinct configurations of the extraction devices. HiSorb probes employ a stainless-steel rod as the support for the sorptive phase [40], whereas the HS-TFSPME-SBSE configuration utilizes a thin-film extraction mesh and a glass-coated stir bar. Previous studies have shown that metal-containing surfaces and metal complexes can facilitate thiol oxidation under certain conditions [16,38,41]. Nevertheless, the present data do not allow direct attribution of the observed sulfur transformations to catalytic effects arising from the stainless-steel substrate. Therefore, any potential contribution of the probe material should be regarded as a plausible but unverified explanation.

The subsequent decline in dimethyl disulfide (c) signal observed with HiSorb (PDMS) following its maximum response, together with the increasing abundance of dimethyl trisulfide (f), is consistent with further sulfur transformations reported for reactive sulfur compounds under thermal analytical conditions [42,43]. However, because reaction pathways were not directly monitored, these observations should be interpreted with caution. In contrast, HS-TFSPME-SBSE exhibited a comparatively stable dimethyl disulfide (c) profile after reaching its maximum at 150 min, suggesting that differences in extraction and desorption conditions may influence the measured sulfur compound distribution. Overall, these findings indicate that the two approaches may differ in their handling of highly reactive VSCs, although dedicated mechanistic studies would be required to determine the exact origin of the observed differences.

### 2.3. Method Validation

Table 1 presents calibration curves for the three extraction methods (HiSorb PDMS, HiSorb DVB/CWR/PDMS and HS-TFSPME-SBSE) used to detect 12 selected VSCs in mango puree, detailing linearity, limit of detection (LOD), limit of quantitation (LOQ), and the linear range. All methods showed good linearity (coefficient of determination (R^2^) > 0.9813), aligning with Huang et al. (2020) [29]. The established linear ranges for most VSCs typically span from 5 µg/kg up to 100 µg/kg or 500 µg/kg, depending on the specific compound. These ranges, often covering a 100-fold difference in concentration (e.g., 5 to 500 µg/kg), provide a sufficiently broad working window for the quantification of VSCs at trace levels commonly encountered in complex fruit matrices such as mangoes. Such dynamic ranges are adequate for capturing the variability in endogenous VSC concentrations and are comparable to those reported in similar studies focused on volatile analysis in mangoes. All methods showed high R^2^ values that indicated robust linearity; however, HS-TFSPME-SBSE can quantify all 12 VSCs with LOD of 0.1–0.6 µg/kg and LOQ of 0.3–1.9 µg/kg, while HiSorb methods showed limitations for certain compounds like diallyl trisulfide. The HiSorb methods also yielded higher LOD (0.3–0.18 µg/kg for PDMS phase and 0.2–0.17 µg/kg for DVB/CWR/PDMS phase) and LOQ (1.0–6.0 µg/kg for PDMS phase and 0.8–5.6 µg/kg for DVB/CWR/PDMS phase) values. These limitations are evidenced by the absence of linear range data for HiSorb in Table 1 due to inability of the HiSorb methods to detect these compounds, potentially affecting trace-level VSCs detection in mango.

The slopes of the calibration curves reflect the methods’ response sensitivity. HiSorb (PDMS) exhibited steeper slopes for lighter molecular weight VSCs, such as methyl mercaptan and dimethyl sulfide, indicating higher response sensitivity. HiSorb (DVB/CWR/PDMS) had slopes from 1778 to 32,131, with a lower slope for dimethyl sulfide compared to HiSorb (PDMS). HS-TF-SPME-SBSE had slopes from 342 to 40,045, showing strong responses to mid-molecular weight VSCs but lower slopes for lighter molecular weight VSCs like methyl mercaptan. HS-TFSPME-SBSE showed balanced slopes across a wide range of VSCs suggest it is more robust for profiling. Although matrix-matched calibration using mango puree was employed during validation to better account for matrix-related influences, matrix effects were not quantitatively evaluated through comparison with solvent-based calibration curves. Consequently, the extent of signal suppression or enhancement arising from the complex mango matrix remains uncertain. Future studies should include dedicated matrix-effect assessments to further establish the quantitative accuracy and robustness of the method across different mango cultivars, tissues, and ripening stages.

In summary, the comparative evaluation of sorptive extraction methods revealed performance differences. Both optimization (Section 2.2) and method validation (Section 2.3) indicated that HS-TFSPME-SBSE and HiSorb (PDMS) exhibited superior extraction efficiency for key VSCs compared to HiSorb (DVB/CWR/PDMS). Notably, HiSorb (DVB/CWR/PDMS) showed lower extraction yields. Potential impurities in the composite coating materials may additionally contribute to these effects by reducing effective sorption sites. Due to these limitations in extraction efficiency, artifact formation, limited linear range and response sensitivity, HiSorb (DVB/CWR/PDMS) was excluded from further analysis.

### 2.4. Comparative Evaluation Under Trace-Level Conditions

The purpose of this section is to compare the extraction efficiencies and reproducibility of HiSorb (PDMS) and HS-TFSPME-SBSE under trace-level conditions. To comprehensively evaluate these methods, two distinct experiments were conducted: (1) detection of VSCs spiked into mango puree at a concentration of 10 μg/kg to simulate natural mango matrices, and (2) direct analysis of VSCs in fresh mango flesh obtained from three mango varieties (Golden Honey, Sindhura and Palmer). This dual approach ensured that both methods were thoroughly evaluated for their sensitivity, reproducibility, and applicability to real mango samples.

#### 2.4.1. Detection of VSCs Spiked at Concentration of 10 μg/kg

The goal of this experiment was to determine which method would exhibit superior sensitivity and reproducibility when detecting low-concentration VSCs.

The comparison reveals that HS-TFSPME-SBSE generally exhibited superior performance over HiSorb (PDMS) across the majority of analytes. Notably, methyl mercaptan was consistently detected by HS-TFSPME-SBSE but was undetectable by HiSorb (PDMS). Additionally, the same was observed for diallyl trisulfide.

In terms of reproducibility, both methods demonstrated satisfactory RSD values under trace-level conditions, with most compounds exhibiting RSDs below 15%. However, HiSorb (PDMS) generally provided better reproducibility than HS-TFSPME-SBSE. This was likely due to the simpler single-phase sorption mechanism of HiSorb (PDMS), which offers consistent adsorption properties. In contrast, the dual-phase of HS-TFSPME-SBSE may introduce greater variability due to more complex sorption and desorption processes.

The results suggest that HS-TFSPME-SBSE is better suited for detecting a broader range of VSCs under trace-level conditions. However, the better reproducibility of HiSorb (PDMS) highlights its potential applicability in specific scenarios where precision is prioritized.

#### 2.4.2. Detection of VSCs in Flesh of Different Mango Varieties

The peak area data for VSCs in fresh mango from three mango varieties (Golden Honey, Sindhura and Palmer), extracted using HS-TFSPME-SBSE and HiSorb (PDMS), provided a direct comparison of the methods’ sensitivity in real sample analysis. In this analysis, no quantification was carried out and the peak areas reported showcase the sensitivity of the methods in detection of different VSCs. This discussion evaluates the sensitivity of methods using peak areas and standard deviations, by comparing the results with spiked sample findings.

The peak area data for mango flesh samples and their standard deviation values in Table 2 reveal significant differences in the sensitivity of HS-TFSPME-SBSE and HiSorb (PDMS) across the mango samples. Despite that HiSorb (PDMS) showed higher peak area in all three mango varieties for dimethyl trisulfide, HS-TFSPME-SBSE consistently yielded higher peak areas compared to HiSorb (PDMS) in three mango varieties for most other VSCs like dimethyl sulfide, dimethyl disulfide and benzothiazole. These higher peak areas indicate that HS-TFSPME-SBSE is more sensitive in the detection of most VSCs in fresh mango. However, HiSorb (PDMS) excels for specific compounds like dimethyl trisulfide, potentially due to oxidative formation. To ensure the accurate profiling of such reactive VSCs, future studies could employ oxidative surrogates to monitor and account for artifact generation during the extraction procedure. From the result in Table 2, high RSD can be observed for some of the values (e.g., 3,4-dimethylthiophene in the Palmer). This can be attributed to the trace-level presence of these volatile sulfur compounds, which are inherently prone to fluctuations arising from sample heterogeneity, matrix effects, and the sensitivity limits of the analytical method, highlighting the need for a sensitive and reproducible method for the detection of these compounds.

### 2.5. PCAs of VSCs in Different Mango Varieties

**Figure 4:**
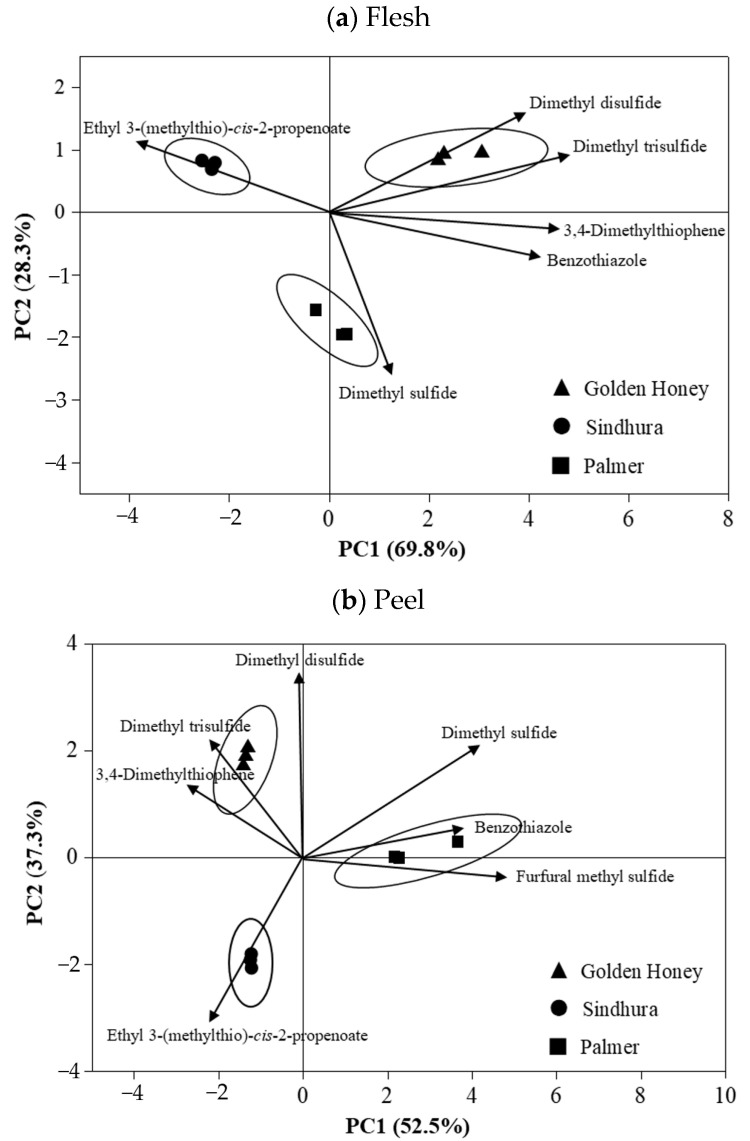
PCA biplots of identified VSCs among three mango cultivars (Golden Honey, Sindhura and Palmer): (**a**) Flesh and (**b**) Peel.

Principal component analysis (PCA) was performed using volatile composition data obtained by the HS-TFSPME-SBSE method (Table S2), which demonstrated a superior extraction capability by capturing most compounds with higher intensity compared to other methods tested. Both mango flesh and peel samples from three varieties (Golden Honey, Sindhura and Palmer) were analyzed separately to explore tissue-specific VSC profiles. The PCA biplots (Figure 4a,b) provided an exploratory visualization of the relationships among mango cultivars based on their VSC compositions. Distinct grouping of the three cultivars was observed in both flesh and peel samples, indicating that the measured VSC profiles differed among varieties. While PCA is not intended to provide statistical evidence of group separation, the observed patterns were consistent with the differences in compound abundance observed. In addition to the 12 selected VSCs, two newly identified compounds—furfuryl ethyl sulfide and ethyl 3-(methylthio)-*cis*-2-propenoate—were detected in fresh mangoes and included in the PCA, which were also reported in previous studies in tropical fruits [11].

For the mango flesh samples (Figure 4a), the first two PCs accounted for 98.1% of the total variance (PC1: 69.8%, PC2: 28.3%). The Golden Honey mango was strongly correlated with dimethyl disulfide and showed moderate associations with dimethyl trisulfide and 3,4-dimethylthiophene, which might contribute to a dominant, sulfur-driven aroma with intense, pungent notes [44]. In contrast, Sindhura mango was primarily associated with ethyl 3-(methylthio)-*cis*-2-propenoate, a sulfur ester known for milder and tropical notes, which can impart a distinct sensory identity emphasizing fruitiness over sulfurous intensity based on literature [11]. Meanwhile, the Palmer mango exhibited a moderate association with dimethyl sulfide which is linked to green and sulfurous notes [45] but did not align closely with any specific VSC loading vectors, indicating the absence of a dominant VSC. Therefore, the result suggested that Palmer mango displayed a balanced distribution of VSCs that likely contributes to a complex, layered aroma, which is consistent with earlier findings [46].

For the mango peels (Figure 4b), the first two PCs explained 89.8% of the total variance (PC1: 52.5%, PC2: 37.3%). Sample separation was also evident, though the distribution of VSCs revealed distinct tissue-dependent profiles. Compared to the flesh, peel of the Golden Honey mango retained a sulfurous signature, with dimethyl disulfide and dimethyl trisulfide remaining prominent contributors to its pungent aroma, indicating a consistent VSC profile. The peel of Sindhura mango also mirrored its flesh profile, with ethyl 3-(methylthio)-*cis*-2-propenoate as the dominant VSC, though overall VSC intensities were lower. In contrast, the peel of Palmer mango diverged significantly from its flesh profile, featuring elevated levels of benzothiazole and furfuryl methyl sulfide—compounds which often reported to associate with thermal, roasted notes that were not dominant in the flesh [47]. This shift highlights a peel-specific volatile profile in Palmer mango, distinguishing it from the more consistent VSC distributions observed in Golden Honey and Sindhura mangoes across different tissues.

The PCA results also highlighted differences between flesh and peel VSC profiles. These tissue-dependent variations are consistent with previous reports demonstrating that mango tissues differ in their biochemical composition, enzyme activity, and precursor availability during ripening [48,49,50,51,52,53,54]. Such factors may contribute to differences in the formation and accumulation of sulfur-containing volatiles. In the peel, presence of polyphenol oxidase (PPO) and peroxidase (POD) might promote the reaction of reactive oxygen species ROS with sulfur precursors, forming heterocyclic VSCs such as benzothiazole in peel of Palmer mango [52,53]. In line with this, furfuryl ethyl sulfide was only detected in the peel of Palmer and Sindhura mangoes, but not in any flesh samples, suggesting this peel-specific origin. Conversely, the flesh exhibits lower PPO and POD activities, resulting in reduced ROS production and a less oxidative environment that limits the formation of complex sulfur heterocycles. This favors the accumulation of simpler VSCs in the flesh, such as methyl mercaptan, dimethyl sulfide and ethyl 3-(methylthio)-*cis*-2-propenoate which form through esterification pathways rather than oxidative reactions [51]. However, the present study did not directly measure enzyme activity, reactive oxygen species, sulfur precursors, or reaction pathways. Therefore, the proposed biochemical mechanisms should be regarded as potential explanations based on existing literature rather than conclusions directly supported by the current dataset.

The PCA results demonstrate that VSC profiles obtained using HS-TFSPME-SBSE contained sufficient information to differentiate mango cultivars and tissues under the conditions investigated. The improved detection of sulfur-containing volatiles achieved by HS-TFSPME-SBSE likely contributed to the enhanced characterization of cultivar-specific profiles. These findings support the suitability of the optimized method for exploratory profiling of trace VSCs in mangoes. Further studies involving comprehensive method validation and mechanistic investigations would be required to establish the specific roles of extraction materials, sorbent geometry, matrix effects, and potential analyte transformations during analysis.

### 2.6. Limitations and Future Perspectives

Despite the high sensitivity and reliability of the developed HS-TFSPME-SBSE method, some limitations should be acknowledged. Firstly, the current study adopts an one-factor-at-a-time (OFAT) approach to simplify method development. This approach does not fully capture potential interaction effects between temperature and time and a more comprehensive optimization strategy—such as a factorial design or response surface methodology—can be carried out in the future to allow simultaneous evaluation of these parameters and may lead to a more robust determination of optimal conditions. Secondly, current quantification relies on internal standard calibration, and although matrix-matched calibration using mango puree was employed to better account for matrix-related influences, matrix effects were not quantitatively evaluated through comparison with solvent-based calibration curves. Consequently, the extent of signal suppression or enhancement arising from the complex mango matrix remains uncertain. Future research should therefore prioritize comprehensive validation through matrix-effect assessment, stable isotope dilution assays (SIDA), and the use of authentic standards to further improve quantitative accuracy and robustness. Thirdly, a spiking level of 5 mg/kg was selected to enable robust evaluation of extraction efficiency and comparative performance among the different extraction tools. While this concentration facilitated reliable assessment of method behavior across a broad dynamic range, it is higher than the trace-level concentrations typically encountered for many VSCs in mango. Therefore, future studies should place greater emphasis on validation at lower, more representative concentrations (e.g., ≤10 μg/kg) to better assess quantitative performance, adsorption behavior, and method robustness under realistic trace-level conditions. Furthermore, the potential loss of volatile sulfur compounds during the desorption process may have affected the accuracy of the measured concentrations and their partial loss under suboptimal desorption conditions could lead to underestimation. Therefore, future work should systematically investigate the influence of desorption parameters (e.g., material of the trapping liner, desorption and cryo-focusing temperatures, and carrier gas flow, etc.) on analyte recovery to improve method robustness. Future research can also investigate PPO/POD activity assays under the exact extraction conditions employed, as well as the use of oxidative surrogates or enzyme inhibitors, to investigate the activity of these enzymes under the extraction conditions of 40 °C and confirm the pathway for the oxidation of thiols. Lastly, although the present study provides detailed analytical profiling of volatile sulfur compounds, their actual contribution to aroma perception was not evaluated. Future research should incorporate Gas Chromatography-Olfactometry (GC-O), odor activity value (OAV) determination, aroma recombination, and sensory evaluation to establish direct relationships between compound abundance and perceived aroma. In addition, a wider range of mango cultivars and ripening stages should be investigated to further understand varietal and developmental influences on sulfur-containing volatiles.

## 3. Materials and Methods

### 3.1. Reagents, Samples and Apparatuses

High performance liquid chromatography (HPLC)-grade methanol (MeOH) was obtained from Fisher Scientific Co. (Waltham, MA, USA). MeOH was chosen as the solvent as it provides high solubility for VSCs.

Twelve authentic reference standards (food grade, purity > 98%) were provided by Mane SEA Pte Ltd. (Singapore), as listed in Table S1. An internal standard, 2-octanol, obtained from Sigma-Aldrich (St. Louis, MI, USA), was also included for quantification. A standard stock solution was prepared by accurately weighing 0.1 g of each pure standard, dissolving in MeOH, and diluting to the 10 mL mark in a volumetric flask. The selection of these 12 VSCs was based on previous studies [6,55], which detailed their presence and concentration in fresh mangoes. C_7_–C_40_ alkane standards were obtained from Sigma-Aldrich.

Mango samples were purchased at the fully ripe stage, characterized by uniform peel coloration and a firm-yet-yielding texture from a local supermarket, with three varieties sourced from Thailand (Golden Honey), India (Sindhura) and Brazil (Palmer). Mango puree was obtained from REDMAN, Singapore. All fresh mangoes were analyzed immediately upon purchase. The mango puree was stored at 4 °C within one month before analysis.

Twister bars (sorbent volume 24 μL, sorbent surface area 154 mm^2^) and TFSPME devices (sorbent volume 33 μL, sorbent surface area 344 mm^2^) were acquired from Gerstel GmbH & Co. KG (Mülheim an der Ruhr, Germany). HiSorb probes (sorbent volume 65 μL, sorbent surface area 96.1 mm^2^) were procured from Markes International Ltd. (Bridgend, UK).

### 3.2. Sample Preparation

A standard stock solution containing 12 VSCs was prepared in MeOH, with each VSC at a concentration of 10 mg/kg. The stock solution was then serially diluted using MeOH and spiked into mango puree to obtain a series of concentrations from 5 to 5000 µg/kg. The purees were thoroughly mixed to ensure homogeneity, thereby simulating the mango matrix environment. Additionally, a 10 mg/kg standard solution of 2-octanol was prepared in MeOH for use as an internal standard to account for variations in instrument response across injections. While comparative evaluation of the extraction devices was based on raw peak areas to reflect absolute capacity, the consistent response of 2-octanol verified the robustness and reliability of the analytical sequence.

Fresh mangoes were peeled, deseeded, and flesh cut into cubes (0.5 cm × 0.5 cm × 0.5 cm) for the pulp and slices (0.5 cm × 0.5 cm × 0.1 cm) for the peel. The pulp and peel analysed were pooled from multiple mango fruits. For each analysis, 3 g of mango puree, pulp, or peel was transferred into a 20 mL glass headspace vial (Gerstel GmbH & Co. KG), generating VSC-spiked mango puree samples alongside fresh mango pulp and peel samples. Each 3 g sample was spiked with 0.15 g of the 2-octanol standard solution.

HS extractions were carried out using the different sorptive devices. The extraction conditions for each of the different devices were adapted and modified based on the approach of Huang et al. (2020) [29]. Prior to use, HiSorb probes, twister bars and TFSPME devices were conditioned at 230 °C for 1 h under a continuous nitrogen gas flow. After each use, they were reconditioned through thermal treatment at 230 °C for 3 h under nitrogen gas. For the HiSorb extraction method, HiSorb probes were inserted through the sealed cap to facilitate static headspace sampling. For twister bar and TFSPME, a metal holder (Gerstel GmbH & Co. KG) was used to hold and expose the tools to the sample headspace. Its clip-based mechanism secures the TFSPME device, while the twister bar is attached via magnetic attraction. For the combined extraction methods, the stir bar and TFSPME device were positioned in the sample headspace together with the same metal holder. The insertion depth was standardized at 3 cm across all trials. After positioning the extraction devices, the vials were placed in a water bath (Thermo Fisher Scientific, Waltham, MA, USA) and subjected to heating for a predefined duration described below. Upon completion of extraction, all devices were carefully removed, wiped with lint-free tissues, and transferred to thermal desorption tubes. After preliminary trials, HiSorb (DVB/CWR/PDMS), HiSorb (PDMS) and HS-TFSPME-SBSE, were selected for method optimization based on performance.

### 3.3. Method Optimization and Validation

Optimization was carried out for extraction temperature and extraction time. Two extraction temperatures (30 °C and 40 °C, at 150 min) and five extraction durations (60, 90, 120, 150, 180 min, under 40 °C) were studied for HiSorb of PDMS and DVB/CWR/PDMS and HS-TFSPME-SBSE using OFAT experimental design. A lower extraction temperature range was intentionally selected to better mimic the conditions under which mango is typically consumed and aroma perception occurs during sensory evaluation. Rather than employing elevated temperatures solely to maximize analyte volatilization, this study aimed to obtain VSC profile that is more representative of the aroma released under realistic consumption conditions. Previous studies have demonstrated that when extraction parameters reflective of actual food consumption conditions are applied in sorptive extraction methodologies, the resulting volatile profiles show improved relevance and correlation to sensory perception and evaluation outcomes [56].

The optimized conditions (HiSorb (PDMS): 40 °C, 120 min; HiSorb (DVB/CWR/PDMS): 40 °C, 120 min; HS-TFSPME-SBSE: 40 °C, 150 min) were then validated. This involved spiking mango puree with a series of 12 VSC standard solutions to achieve final concentrations typically ranging from 5 µg/kg to 500 µg/kg (specific ranges for each compound and method are detailed in Table 1. These concentration levels were chosen to establish key validation parameters such as linearity, LOD, and LOQ relevant for trace VSC analysis in mangoes. A higher spiking concentration (5 mg/kg) was used during comparison of different sorptive extraction methods to ensure sufficient analytical response, whereas a lower concentration (5–500 µg/kg) was applied in sequence method optimization to better reflect more representative levels and to minimize potential sorbent overloading effects.

### 3.4. Sample Introduction

After extraction, each HiSorb probe was transferred individually into a separate thermal desorption (TD) tube, while both the twister bar and TFSPME device from the same sample (for the combined extraction method) were placed into a single TD tube. The TD tubes were then subjected to thermal desorption using an autosampler (Gerstel GmbH & Co. KG) integrated with a GC system. The autosampler included a multi-purpose sampler (MPS) with a tube tray, a thermal desorption unit (TDU-2), and a cooled injection system (CIS-4). These components were connected to a cryo-static cooling device (CCD2) to ensure precise temperature control.

Thermal desorption in the TDU-2 followed a programmed temperature sequence: an initial hold at 30 °C for 1.5 min, a ramp to 240 °C at 100 °C/min, followed by a 5 min hold at 240 °C. The desorption flow was maintained at 50 mL/min in splitless mode. The released compounds were transferred to the CIS-4 system via a transfer line set at 280 °C and cryo-focused at −10 °C on a Tenax TA-packed liner positioned in the CIS-4 port. Subsequently, the CIS-4 inlet temperature increased from −10 °C to 240 °C at 10 °C/min, with a 5 min hold at the final temperature to facilitate splitless transfer of the analytes onto the analytical column.

### 3.5. GC-Q-TOF/MS Analysis

Analysis was carried out on an Agilent 7890B GC system coupled with an Agilent 7250 Accurate-Mass quadrupole time-of-flight mass spectrometer (Q-TOF/MS) (Agilent, CA, USA). Compound separation was achieved on an HP-INNOWax column (60 m × 0.25 mm × 0.25 µm) (Agilent, Santa Clara, CA, USA). The column oven temperature was initially set at 50 °C for 5 min, then increased to 240 °C at 5 °C/min, where it was held for 40 min. Helium was used as the carrier gas at a constant flow rate of 1.2 mL/min. Alkane standards were analyzed under the same GC separation condition to determine the linear retention index (LRI) of each compound.

### 3.6. Data Analysis

Data processing was performed using Agilent MassHunter Workstation Quantitative Analysis (B.10.00). Spectral peaks were extracted using extracted ion chromatogram (EIC) deconvolution workflows, with mass-to-charge ratios (*m*/*z*) selected based on the quantitative ion of each VSC. Compound identification was conducted by comparison of their mass spectra and LRIs against that of NIST library (2014) and authentic standards. All analyses were conducted in triplicate, and mean values along with standard deviations were calculated using Microsoft Excel (2024, Microsoft Corporation, Redmond, WA, USA). One-way ANOVA was calculated using SPSS Statistics (IBM, version 25.0.0) based on 95% confidence level to assess whether significant differences existed among the group means for comparison of the different extraction tools and method development. Due to the limited number of replicates, formal tests of normality and homogeneity of variance were considered to have limited statistical power; therefore, ANOVA results should be interpreted as supporting comparative trends rather than constituting a comprehensive statistical validation. The identified VSCs in mangoes were used to generate PCA scores and loadings plots with Origin 2022 SR1 (OriginLab, Northampton, MA, USA).

## 4. Conclusions

In conclusion, by utilizing mango as a complex matrix model, a methodological framework demonstrating how material selection (to prevent thiol oxidation) and device geometry (surface area for kinetics and volume for capacity) interact with temperature and time to influence VSC detection was developed. Optimized conditions were identified as 120 min at 40 °C for HiSorb (PDMS) and 150 min at 40 °C for HS-TF-SPME-SBSE. Overall, HS-TF-SPME-SBSE exhibited improved analytical performance compared to HiSorb (PDMS), showing higher sensitivity and broader VSC coverage, particularly for low-abundance and labile sulfur compounds. Method validation further demonstrated good linearity and low detection limits, supporting its suitability for trace-level analysis. Beyond methodological comparison, the findings provide practical insights into how extraction conditions, sorptive materials, and matrix interactions influence the analytical performance of sorptive extraction techniques, offering guidance for future optimization and application in other complex food matrices. However, because the optimization was conducted using an OFAT approach, potential interactions among variables were not investigated. Therefore, the selected conditions should be regarded as the most favorable within the experimental space examined rather than a definitive global optimum. Future studies employing factorial experimental design or response surface methodology may provide a more comprehensive evaluation of factor interactions and further refine the extraction conditions.

Multivariate analysis (PCA) enabled clear discrimination of tissue-specific and cultivar-dependent VSC profiles among different mango varieties, highlighting distinct compositional patterns. While differences between peel and flesh were discussed in relation to potential biochemical processes, these interpretations remain tentative and require further experimental validation. These findings provide useful insights into the analysis of reactive and low-concentration volatiles in complex botanical matrices and demonstrate promising performance of the method across the matrices examined. Such advancements will further support the development of robust, solvent-free analytical approaches for applications in food flavor analysis, quality control, and authentication. Future work should focus on improving design of experiments, quantitative accuracy, as well as validating proposed mechanisms through targeted experiments.

## Figures and Tables

**Table 1:** (**a**). Calibration curves for volatile sulfur compounds (VSCs) spiked in mango puree, extracted by HiSorb (PDMS) (40 °C, 120 min), HiSorb (DVB/CWR/PDMS) (40 °C, 120 min) and HS-TFSPME-SBSE (40 °C, 150 min). (**b**). Detection limits for VSCs spiked in mango puree, extracted by HiSorb (PDMS) (40 °C, 120 min), HiSorb (DVB/CWR/PDMS) (40 °C, 120 min) and HS-TFSPME-SBSE (40 °C, 150 min).

(a)															
No.	Compound	Calibration Curve													
		Linear Equation				Range (µg/kg)						*R* ^2 1^			
		HiSorb (PDMS)	HiSorb (DVB/CWR/PDMS)		HS-TFSPME-SBSE	HiSorb (PDMS)		HiSorb (DVB/ CWR/ PDMS)		HS-TFSPME-SBSE		HiSorb (PDMS)	HiSorb (DVB/ CWR/ PDMS)		HS-TFSPME-SBSE
1	Methyl mercaptan	-	-		*y* = 342*x* + 2806	-		-		5 to 100		-	-		0.9927
2	Dimethyl sulfide	*y* = 4123*x* + 6239	*y* = 597*x* − 4023		*y* = 16,021*x* + 8618	5 to 100		10 to 100		5 to 100		0.9921	0.9958		0.9988
3	Dimethyl disulfide	*y* = 27,756*x* + 42,817	*y* = 10,998*x* − 13,216		*y* = 24,322*x* − 231,774	5 to 500		5 to 500		5 to 500		0.9973	0.9876		0.9989
4	Diallyl sulfide	*y* = 14,323*x* +14,397	*y* = 9702*x* − 19,139		*y* = 22,417*x* − 111,857	5 to 500		5 to 500		5 to 500		0.9949	0.9949		0.9964
5	3,4-Dimethylthiophene	*y* = 32,131*x* − 37,926	*y* = 24,424*x* + 5640		*y* = 40,045*x* − 9198	5 to 500		5 to 500		5 to 500		0.9925	0.9925		0.9999
6	Dimethyl trisulfide	*y* = 27,614*x* + 822	*y* = 30,123*x* − 54,778		*y* = 35,376*x* + 6238	5 to 300		5 to 300		5 to 500		0.9883	0.9813		0.9999
7	4-Mercapto-4-methylpentan-2-one	*y* = 4490*x* + 1526	*y* = 1019*x* + 16,893		*y* = 5102*x* − 83,063	5 to 500		5 to 500		5 to 500		0.9880	0.9984		0.9981
8	Diallyl disulfide	*y* = 10,682*x* − 53,438	*y* = 8301*x* − 8177		*y* = 8554*x* + 38,645	5 to 500		5 to 500		5 to 500		0.9933	0.9923		0.9994
9	Methyl propyl trisulfide	*y* = 16,042*x* − 59,538	*y* = 15,953*x* − 39,788		*y* = 11,000*x* + 157,198	5 to 500		5 to 500		5 to 500		0.9849	0.9935		0.9961
10	Diallyl trisulfide	-	-		*y* = 688*x* + 32,860	-		-		5 to 500		-	-		0.9941
11	Benzothiazole	*y* = 12,863*x* − 1655	*y* = 18,747*x* − 35,830		*y* = 23,276*x* − 109,524	5 to 500		5 to 500		5 to 500		0.9915	0.9936		0.9997
12	Furfuryl disulfide	*y* = 1778*x* − 18,230	*y* = 2021*x* − 94		*y* = 757*x* + 20,373	10 to 300		5 to 300		5 to 500		0.9868	0.9935		0.9932
(**b**)															
**No.**	**Compound**		**Detection Limit (µg/kg) ^2^**												
			**LOD**						**LOQ**						
			**HiSorb (PDMS)**	**HiSorb (DVB/CWR/PDMS)**			**HS-TFSPME-SBSE**		**HiSorb (PDMS)**		**HiSorb (DVB/CWR/PDMS)**			**HS-TFSPME-SBSE**	
1	Methyl mercaptan		-	-			0.6		-		-			1.9	
2	Dimethyl sulfide		0.9	1.7			0.2		2.9		5.6			0.5	
3	Dimethyl disulfide		0.9	0.4			0.4		3.0		1.2			1.3	
4	Diallyl sulfide		0.8	1.2			0.3		2.7		3.9			1.0	
5	3,4-Dimethylthiophene		0.3	0.5			0.2		1.0		1.7			0.7	
6	Dimethyl trisulfide		0.4	0.9			0.2		1.3		2.9			0.6	
7	4-Mercapto-4-methylpentan-2-one		0.8	1.0			0.3		2.6		3.4			1.0	
8	Diallyl disulfide		0.9	1.4			0.3		3.0		4.6			1.0	
9	Methyl propyl trisulfide		0.8	0.9			0.4		2.5		3.3			1.2	
10	Diallyl trisulfide		-	-			0.6		-		-			1.8	
11	Benzothiazole		0.3	0.2			0.1		0.9		0.8			0.3	
12	Furfuryl disulfide		1.8	0.9			0.5		6.0		3.3			1.6	

^1^ *R*^2^: Coefficient of determination, indicating the goodness of fit for the calibration curve, with values closer to 1 indicate better linearity. ^2^ Detection limit: Calculated as: LOD (Limit of detection) = 3 × (σ/S), LOQ (Limit of quantitation) = 10 × (σ/S), where σ is the standard deviation of replicate responses at the lowest concentration within the linear range, and S is the slope of the calibration curve.

**Table 2:** Extraction profiles (peak area) of identified VSCs in mango flesh using HiSorb (PDMS) (40 °C, 120 min) and HS-TFSPME-SBSE (40 °C, 150 min).

No.	Compound	LRI		Peak Area						Identification methods ^3^
				Golden Honey		Sindhura		Palmer		
		Ref. ^1^	Expt. ^2^	HiSorb (PDMS)	HS-TFSPME-SBSE	HiSorb (PDMS)	HS-TFSPME-SBSE	HiSorb (PDMS)	HS-TFSPME-SBSE	
1	Dimethyl sulfide	754	742	40,428 ± 2412 ^b^	151,677 ± 5157 ^a^	36,817 ± 1237 ^b^	98,220 ± 3438 ^a^	77,155 ± 2062 ^b^	333,729 ± 9033 ^a^	LRI, MS, STD
2	Dimethyl disulfide	1077	1085	286,921 ± 5551 ^b^	331,497 ± 1181 ^a^	125,924 ± 2776 ^b^	154,819 ± 4429 ^a^	65,132 ± 5551 ^b^	96,233 ± 1476 ^a^	LRI, MS, STD
3	3,4-Dimethylthiophene	1252	1269	7169 ± 1250 ^b^	24,388 ± 2155 ^a^	19,810 ± 3213 ^a^	10,274 ± 4078 ^a^	26,378 ± 3213 ^a^	18,056 ± 5078 ^a^	LRI, MS, STD
4	Dimethyl trisulfide	1383	1389	100,423 ± 12,523 ^b^	70,840 ± 4742 ^a^	56,050 ± 7523 ^b^	13,228 ± 1613 ^a^	36,720 ± 3330 ^b^	12,466 ± 731 ^a^	LRI, MS, STD
5	Benzothiazole	1958	1998	79,818 ± 1286 ^b^	147,297 ± 5703 ^a^	12,594 ± 1286 ^b^	33,243 ± 1901 ^a^	24,071 ± 1286 ^b^	93,064 ± 2901 ^a^	LRI, MS, STD
6	Ethyl 3-(methylthio)-*cis*-2-propenoate	1726	1728	-	-	15,642 ± 1071 ^b^	27,394 ± 2547 ^a^	-	-	LRI, MS

Values for each compound are presented as mean ± standard deviation. “-” Means compounds were not detected. ^a,b^ Different lower-case letters denote that values are significantly different (*p* < 0.05) within the row. ^1^ Ref. LRI: Reference retention index values from the literature: NIST library version 2.2. ^2^ Expt. LRI: Linear retention index on an HP-INNOWax column relative to C_7_–C_40_ alkane standards. ^3^ Identification methods: LRI, comparison of experimental to reference retention indices; MS, comparison with mass spectrum of the compound in the NIST library version 2.2; and STD, comparison with authentic standards.

## Data Availability

The original contributions presented in this study are included in the article. Further inquiries can be directed to the corresponding author.

## Supplementary Materials

The following supporting information can be downloaded at: https://www.mdpi.com/article/10.3390/molecules31132276/s1. Table S1: Selected volatile sulfur compounds (VSCs) and their validation parameters; Table S2: Extraction profiles of identified VSCs in flesh and peel among three mango cultivars using HS-TFSPME-SBSE (40 °C, 150 min).

## Abbreviations

The following abbreviations are used in this manuscript:

CCD	Cryo-static cooling device
CIS	Cooled injection system
CWR	Carbon wide range
DVB	Divinylbenzene
EIC	Extracted ion chromatogram
GC	Gas chromatography
H_2_O_2_	Hydrogen peroxide
HPLC	High performance liquid chromatography
HS-TFSPME-SBSE	Headspace thin-film solid-phase microextraction-stir bar sorptive extraction
LOD	Limits of detection
LOQ	Limits of quantification
*m*/*z*	Mass-to-charge ratios
MeOH	Methanol
MPS	Multi-purpose sampler
O	Olfactometry
OFAT	One-factor-at-a-time
PCA	Principal component analysis
PDMS	Polydimethylsiloxane
POD	Peroxidase
PPO	Polyphenol oxidase
Q-TOF/MS	Quadrupole time-of-flight mass spectrometer
R^2^	Coefficient of determination
ROS	Reactive oxygen species
RSD	Relative standard deviation
SBSE	Stir-bar sorptive extraction
SIDA	Stable Isotope Dilution Assays
SPME	Solid-phase microextraction
TD	Thermal desorption
TDU	Thermal desorption unit
TFSPME	Thin-film solid-phase microextraction
VOCs	Volatile organic compounds
VSCs	Volatile sulfur compounds
