# Supplementary material for: Detection of Volatile Sulfur Compounds in Mangoes Using Sorptive Extraction Methods
Source: Molecules. 2026 Jun 29;31(13):2276. doi: 10.3390/molecules31132276 (PMC13363637; doi:10.3390/molecules31132276)
Supplement: Supplementary file 1 [file molecules-31-02276-s001.zip › molecules-4358682-supplementary.pdf]

Supplementary information

Table S1. Selected volatile sulfur compounds (VSCs) and their validation parameters.

No.	Compound	CAS	Formula	Molecular Mass [2] (g/mol)	Log *P* [3]	Quantitative ion [4] (*m/z*)
1	Methyl mercaptan	74-93-1	CH_4S	48.11	0.5	46.9955
2	Dimethyl sulfide	75-18-3	C_2H_6S	62.14	0.9	62.0190
3	Dimethyl disulfide	624-92-0	$C_2H_6S_2$	94.20	1.8	93.9911
4	Diallyl sulfide	592-88-1	$C_6H_{10}S$	114.21	2.2	44.9799
5	3,4-Dimethylthiophene	632-15-5	C_6H_8S	112.19	2.3	111.0268
6	Dimethyl trisulfide	3658-80-8	$C_2H_6S_3$	126.30	1.3	125.9632
7	4-Mercapto-4-methylpentan-2-one	19872-52-7	$C_6H_{12}OS$	132.23	0.8	43.0184
8	Diallyl disulfide	2179-57-9	$C_6H_{10}S_2$	146.30	2.2	41.0391
9	Methyl propyl trisulfide	17619-36-2	$C_4H_{10}S_3$	154.30	2.2	153.9945
10	Diallyl trisulfide	2050-87-5	$C_6H_{10}S_3$	178.30	2.6	73.0112
11	Benzothiazole	95-16-9	C_7H_5NS	135.19	2.0	135.0143
12	Furfuryl disulfide	4437-20-1	$C_{10}H_{10}O_2S_2$	226.30	2.1	81.0340

[1] LRI: Linear retention index on an HP-INNOWax column relative to C_7-C_{40} alkane standards.

[2] Molecular mass values were obtained from PubChem.

[3] Log *P*: The logarithm of the partition coefficient between octanol and water; values were obtained from PubChem.

[4] Quantitative ion: The most intense ion in the mass spectrum of a compound, corresponding to the highest abundance.

Table S2. Extraction profiles (peak area) of identified VSCs in flesh and peel among three mango cultivars using HS-TFSPME-SBSE (40 °C, 150 min).

No.	Compound	Golden Honey		Sindhura		Palmer		Identification [1]
		Flesh	Peel	Flesh	Peel	Flesh	Peel	
1	Dimethyl sulfide	151677 ± 5157[a]	127461 ± 2908[b]	98220 ± 3438[a]	58332 ± 2711[b]	333729 ± 9033[a]	214123 ± 7960[b]	LRI, MS, STD
2	Dimethyl disulfide	331497 ± 11811[a]	104744 ± 8468[b]	154819 ± 4429[a]	-	96233 ± 1476[a]	56175 ± 1298[b]	LRI, MS, STD
3	3,4-Dimethylthiophene	24388 ± 2155[a]	17653 ± 1570[b]	10274 ± 4078[a]	8458 ± 518[a]	18056 ± 5078[a]	-	LRI, MS, STD
4	Dimethyl trisulfide	70840 ± 4742[a]	12341 ± 833[b]	13228 ± 1613[a]	4231 ± 193[b]	12466 ± 731[a]	-	LRI, MS, STD
5	Benzothiazole	147297 ± 5703[a]	61471 ± 2781[b]	33243 ± 1901[a]	27941 ± 1235[b]	93064 ± 2901[a]	332029 ± 9820[b]	LRI, MS, STD
6	Ethyl 3-(methylthio)-*cis*-2-propenoate	-	-	27394 ± 2547[a]	92793 ± 7449[b]	-	-	LRI, MS
7	Furfuryl methyl sulfide	-	-	-	29771 ± 955[a]	-	31209 ± 978[a]	LRI, MS

Values for each compound are presented as mean ± standard deviation. "-" Means compounds were not detected.

[a,b] Different lower-case letters denote that values are significantly different ($p < 0.05$) within the row.

[1] Identification methods: "LRI", comparison of experimental to reference retention indices; "MS", comparison with mass spectrum of the compound in the NIST library; and "STD", comparison with authentic standard
